# Trends and Correlates of Overweight among Pre-School Age Children, Adolescent Girls, and Adult Women in South Asia: An Analysis of Data from Twelve National Surveys in Six Countries over Twenty Years

**DOI:** 10.3390/nu11081899

**Published:** 2019-08-14

**Authors:** Kassandra L. Harding, Victor M. Aguayo, Patrick Webb

**Affiliations:** 1 Yale School of Public Health, Yale University, New Haven, CT 06515, USA; 2 Friedman School of Nutrition Science and Policy, Tufts University, Boston, MA 02111, USA; 3 United Nations Children’s Fund (UNICEF) Programme Division, New York, NY 10017, USA

**Keywords:** South Asia, overweight, malnutrition, children, adolescent girls, women

## Abstract

Overweight has become a global pandemic and is associated with a rise in diet-related non-communicable diseases and associated co-morbidities. Most of the world’s undernourished people live in South Asia, yet the number of overweight and obese individuals in this region is growing. This study explores trends and correlates of overweight among pre-school age children, adolescent girls, and adult women in South Asia. Using pooled data from 12 national surveys in six countries, generalized linear mixed models were run to analyze relationships. Overweight children had significantly higher odds than non-overweight children of having an overweight mother (Adjusted Odds Ratio: 1.34, *p* < 0.01). Overweight adolescent girls were more likely to come from a wealthier household (Adjusted Prevalence Ratio (APR): 2.46, *p* < 0.01) in an urban area (1.74, *p* < 0.01), and have formal education (1.22, *p* < 0.01), compared to non-overweight girls. Similar relationships were seen among overweight vs. non-overweight adult women. In Bangladesh, India, and Nepal, overweight among girls and women increased over time, while differentials associated with household wealth, urban residence, and formal education attenuated over time. Overweight and obesity are becoming more prevalent across South Asia in a context of persisting undernutrition. Once a condition of the wealthier, more educated and urban, rates of overweight are increasing among poorer, less educated, and rural women. This requires immediate attention to ‘multi-use’ policies and programmes.

## 1. Introduction

Overweight is a growing pandemic that increasingly affects low- and middle-income countries (LMICs). Currently, two-thirds of the globe’s obese people reside in LMICs. Governments in these countries are now challenged to design and implement effective policies, strategies and programmes that respond to the co-existing threats of undernutrition—including micronutrient deficiencies as well as overweight and obesity [1,2]. For example, South Asia is home to an estimated 58.7 million stunted preschoolers, which represents approximately 2 in 5 of all stunted preschoolers worldwide, but also to 5.2 million children aged 0 to 59 months who are overweight (3.1%)—representing 14% of the global burden of overweight among preschoolers [3]. The number of preschool-age children in South Asia classified as overweight has risen from 4.4 million in 2000 to 5.2 million in 2018 [3]. Among adult women, the age-standardized prevalence of overweight was 22.5% in 2013 [1].

The high prevalence of overweight and obesity has significant health and economic implications for the region. Diabetes and cardiovascular disease represent two prominent comorbidities of obesity, and such metabolic risks associated with obesity are known to be greater among Asians than for other ethnic groups [4]. Indeed, a systematic review specific to the Asia-Pacific region found that overweight and obesity accounted for almost 10% of some countries’ healthcare expenditure [5]. Furthermore, the implications of childhood and adolescent overweight and obesity include an increased risk for premature mortality [6].

Individual behaviors, such as low physical activity, increased leisure time spent using electronic screens, and regular intake of fast- and calorically-dense foods, plus socioeconomic status and family history of obesity, are known to be important risk factors for overweight [7]. Such findings were reported in a comprehensive review of childhood obesity across low-income countries, in which endocrine and genetic factors were also discussed as determinants of overweight and obesity among school-aged children and adolescents [8]. Higher education and socioeconomic status are consistently associated with overweight and obesity, although the relationship varies by context. In a cross-sectional analysis among adults in low- and middle-income countries, more years of formal education, residence in urban settings, and higher socioeconomic status (SES) were associated with higher prevalence of obesity [9].

By contrast, overweight and obesity have been associated with lower SES in high-income countries. Popkin et al. reported that the relationship between overweight and SES in low- and middle-income countries has shifted over time and across gross national product (GNP) of countries, such that the poor are also affected [10,11,12,13,14].

It is therefore important to understand the pattern and determinants of the still-evolving epidemic of overweight across South Asia. The objectives of this paper are three-fold: (1) to examine relationships between specific possible determinants of children’s weight-for-height *z*-score (WHZ), adolescent girls’ body mass index (BMI), and women’s BMI, and the respective outcome; (2) to assess whether these relationships differ across countries in South Asia; and (3) to evaluate how these relationships may have changed over time in countries where time-trend analysis is feasible.

## 2. Methods

### 2.1. Datasets

This analysis was based on data from recent nationally-representative health, nutrition and demographic surveys conducted in South Asia, including—in the case of Bangladesh, India and Nepal—multiple surveys. Specifically: The Afghanistan National Nutrition Survey (NNS) 2013; Bangladesh Demographic and Health Survey (DHS) 2014, 2007 and 1997; India DHS 2016, 2006 and 1999; Maldives DHS 2009; Nepal DHS 2016, 2006, and 1996; and Pakistan DHS 2013.

All surveys included in this analysis used multistage cluster sampling methodology, though there was some variation in survey methods by country. Households from select enumeration areas (i.e., clusters) were selected for participation, some surveys implemented stratification, and all included sample weights in the dataset. Sample weights for Afghanistan were not provided on the same scale as those used in the DHSs and weights were not included in the models for this analysis. Detailed study designs have been published previously in each survey’s final report [15,16,17,18,19,20,21,22,23,24,25,26]. In previously published analysis, we took a similar approach to merge similar datasets representing South Asia [27].

Data from each of the selected surveys were imported into Stata14.1 (Stata Corp., College Station, TX, USA), where datasets by participant group were merged together for analysis. Twelve datasets from six countries were included in this analysis with dates ranging from 1996 to 2016.

### 2.2. Analytic Samples

This study included three age groups: pre-school aged children (boys and girls) aged 0–59 months, adolescent girls aged 15 to 19 years, and adult women aged 20 to 49 years. Due to a lack of consistent nationally-representative data on adolescents in the region, we limited our sample of adolescents to older adolescent girls, a subset of the DHS sample of “women of reproductive age: 15 to 49 years old. Children were included in the analysis if they had a plausible WHZ (i.e., ≥−5 and ≤5) and had a non-pregnant mother for whom anthropometric measures were recorded. Adolescent girls and adult women were included if they were not pregnant and had a plausible BMI (i.e., >12 and <60 kg/m^2^).

### 2.3. Child Variables

Anthropometric measures of height and weight were measured for each child, one to three times depending on the survey. The sex of the child was recorded, and age was calculated based on date of birth or based on maternal report. WHZ and height-for-age *z*-score (HAZ) were calculated for each child using the WHO growth reference standards [28]. Appropriate *z*-score variables existed in the DHS datasets and were calculated for the Afghanistan NNS dataset using *zscore06* [29]. Wasting (WHZ < −2), stunting (HAZ < −2) and overweight (WHZ > 2) were defined using corresponding *z*-scores [28].

Indicators of optimal complementary feeding practices as defined by the World Health Organization (WHO) include: (1) children aged 6 to 23 months meeting the minimum meal frequency (MMF) of receiving semi-solid or soft foods; (2) children aged 6 to 23 months meeting the minimum diet diversity (MDD) of consuming 4 or more of the 7 food groups on the day prior to the survey; and (3) children aged 6 to 23 months meeting the minimum acceptable diet (MAD), an indicator that combines breastfeeding/milk-based feeds, meeting the MMF and MDD [30,31]. These indicators were derived from maternal recall questions regarding breastfeeding and the provision and frequency of complementary feeds by food groups. Because the infant and young child feeding (IYCF) module for the DHS differed in earlier years, these variables were only built with data from the most recent survey for each country. In Afghanistan, adequate data was not available to determine MDD and MAD.

### 2.4. Adolescents’ and Women’s Variables

Adolescent girls’ and women’s age in years was available in most datasets, but it was only available for a subsample of women in Afghanistan. Typically, a girl or woman was asked whether she was married, currently pregnant, number of times she had given birth, and level of formal education completed; the latter was dichotomized into no formal education versus formal education. Height and weight were measured and BMI was calculated and categorized into underweight (<18.5 kg/m^2^), normal (18.5–24.9 kg/m^2^), overweight (≥25.0 kg/m^2^), and obese (≥30.0 kg/m^2^) according to the International Classifications [32]. There has been discussion on the most appropriate BMI cut-off for overweight in Asian populations and ≥23.0 kg/m^2^ has been considered. Thus, we calculated the prevalence of overweight using this cut-off [33] as well; however, we use the globally recognized cut-off of ≥25.0 kg/m^2^ for all models. For girls aged 15 to 19 years old, we present BMI values based on the formula: (weight in kg)/(height in meters * height in meters). However, we also report the prevalence of underweight, overweight, and obesity based on International Classifications as well as those proposed by the International Obesity Task Force (IOTF) sex and age-adjusted values for adolescent girls [34]. Short stature was defined as height <145 cm.

### 2.5. Household and Environmental Variables

A wealth index was created for each country separately using principal component analysis based on household asset ownership, such as bicycles, televisions, and type of water access [35,36]. The country-specific index and index quintiles were available in each DHS dataset. For Afghanistan from which we worked with the National Nutrition Survey data, we derived wealth index and quintiles using the methods from the corresponding final report [22]. Wealth quintiles, the standard DHS representation of wealth, were dichotomized into “poor”, the lower two quintiles, and “not poor”, the upper three quintiles. A distinction of urban or rural setting was recorded for each observation. Each country had distinct sub-regions: 34 provinces in Afghanistan, 7 divisions in Bangladesh, 29 states in India, 6 geographic regions of Maldives, 3 ecological zones in Nepal and 6 provinces and regions of Pakistan.

### 2.6. Statistical Analysis

With pooled data from the most recent year for each country, we used generalized linear mixed models to evaluate relationships among factors associated with WHZ and overweight among children, and with BMI and overweight among adolescent girls and adult women. Predetermined covariates were included in the models and standard errors were adjusted for the clustered design of the surveys. Specifically, models evaluating child outcomes of WHZ and overweight, adjusted for child’s age, child’s sex, mother’s age, mother’s formal education status, household wealth, urban residence. Among women, model variables included individual’s age, formal education status, household wealth, and urban residence; and among adolescents included formal education status, household wealth, and urban/rural residence. Adjusted beta coefficients from models evaluating continuous outcomes (i.e., WHZ and BMI), and adjusted odds ratios (AORs) and adjusted prevalence ratios (APRs) from models evaluating rare (prevalence ≤10% in the overall sample) and not rare (prevalence >10% in the overall sample) dichotomous outcomes (i.e., overweight), respectively, were reported [37,38].

For child WHZ and overweight, we examined four key variables: mother’s overweight status, meeting the MMF, the MDD, and the MAD. For both adolescent girls and women, the three key variables tested as correlates to each overweight and BMI were: any formal education, being wealthier, and urban residency. For each key variable listed above, we tested the interaction with country to determine how relationships differed by country. If country was a significant effect modifier (*p* < 0.10), the model was stratified by country and country sub-regions were included as a covariate in the model.

To examine the time trend in these relationships, we pooled data from three survey years for each Bangladesh (1997, 2007 and 2014), India (1999, 2006 and 2016) and Nepal (1996, 2006 and 2016). We re-ran the previously described analysis for each of these three countries separately, and examined the interaction between the key variables previously described and survey year. If survey year was a significant effect modifier (*p* < 0.10), the model was stratified by survey year.

### 2.7. Ethical Considerations

Each survey’s ethical procedures were published in the respective survey reports; informed consent was provided by study participants. For our analysis, we used de-identified datasets.

## 3. Results

A total of 309,157 children aged 0–59 months, 155,306 adolescent girls aged 15–19 years, and 762,017 adult women aged 20–49 years were included in our analytical sample pooled across six countries, 12 national surveys and 20 years (Table 1). In addition, the most recent surveys for six countries (2006 to 2016) were pooled, allowing us to build an analytic sample comprising 231,121 pre-school age children, 123,990 adolescent girls and 575,052 adult women.

### 3.1. Pre-School Aged Children

Mean (± SD) WHZ and HAZ among pre-school aged children across the six countries from recent survey years were −0.91 ± 1.40 and −1.46 ± 1.68, respectively, and the prevalence of overweight was 2.6%. Children of overweight mothers had a significantly higher mean WHZ (−0.55 ± 1.37) compared with children of mothers not overweight (−0.98 ± 1.40) after adjusting for covariates (*p* < 0.01) and had greater odds of being overweight themselves (AOR (Standard Error (SE)): 1.34 (0.05); *p* < 0.01). The difference in mean WHZ by maternal overweight remained independently significant in each country (*p* < 0.05). Increased odds of a child being overweight if their mother was overweight was significant in India, Maldives, Nepal and Pakistan (Table 2).

**Figure 1:**
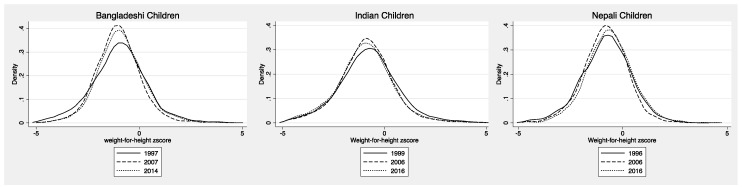
Distribution of weight-for-height *z*-score (WHZ) of children 0 to 59 months of age in Bangladesh, India and Nepal over time.

We tested the relationship between child WHZ and maternal overweight was examined over time. Figure 1 depicts the WHZ distribution among Bangladeshi, Indian, and Nepali children across the three survey time points observed for each country. Overall in Bangladesh, India and Nepal, children of overweight mothers had significantly higher mean WHZs compared with children of non-overweight mothers (Table S1). This relationship differed significantly over time in Bangladesh and India. For example, the mean WHZ among children of overweight mothers was higher than among children with mothers who were not overweight in India at each time-point, yet this difference decreased overtime. In Bangladesh, the difference in mean WHZ among children of overweight vs. non-overweight mothers was not significant in 1997. In contrast, in 2007 and 2014, children of overweight mothers had a significantly higher mean WHZ compared to those children of non-overweight mothers (Table S1).

In the pooled, six country sample, for any given indicator of complementary feeding—MMF, MDD and MAD—meeting the indicator was independently associated with a higher mean WHZ among children 6 to 23 months old compared with children who did not meet the indicator in question. Furthermore, children who met MDD and MAD, had a higher likelihood of being overweight than children who did not meet the MDD and MAD (1.28 (0.07); *p* < 0.01 and 1.19 (0.10); *p* < 0.05 respectively). (Table 3). Country did not significantly modify these relationships.

### 3.2. Adolescent Girls

The sample of adolescent girls included 15- to 19-year-old girls; 7.5% of them had no formal education and ~13% were married (Table 1). Using the IOTF BMI adjustments for sex and age, 4.6% of the adolescent girls were overweight based on the cutoff of ≥25.0 kg/m^2^ and 11.6% were overweight using the cutoff of ≥23.0 kg/m^2^. In adjusted models, mean BMI and the prevalence of overweight (BMI ≥25.0 kg/m^2^) were significantly higher among girls of non-poor households (3rd to 5th wealth quintiles) and among girls of urban households, while formal education was significantly associated with the prevalence of overweight but not associated with mean BMI (Table 4). The associations between education and wealth with mean BMI were modified by country because the relationship of the two exposure variables (education and wealth) with BMI differed in magnitude across some countries. When stratified by country, urban residence was significantly associated with higher odds of being overweight among adolescent girls in India only (AOR (SE): 1.77 (0.06); *p* < 0.01), and household wealth was significantly associated with higher odds of being overweight in Bangladesh (2.73 (0.71); *p* < 0.01), India (2.27 (0.09); *p* < 0.01), Nepal (2.07 (0.70); *p* < 0.05) and Pakistan (16.68 (20.90); *p* < 0.05) (Table 4).

The likelihood of being overweight increased significantly among adolescent girls with survey year in Bangladesh, but not in India or Nepal (Table S2); and the rate of change in overweight and obesity in recent years among these three countries was highest in Bangladesh at 0.14 and 0.44 percentage points per year, respectively, between 2007 and 2014 (Table S3). Survey year was not a significant effect modifier for any key factors in relation to overweight among adolescent girls in Bangladesh, India or Nepal, thus the relationship between key factors and overweight did not differ by survey year (Table S2).

### 3.3. Adult Women

Adult women in this sample were on average 33.0 ± 8.4 years old and 34% of them had no formal education. Approximately 10% of the women were of short stature (<145 cm), 18% were underweight (BMI ≤18 kg/m^2^) and 22% were overweight (BMI ≥25.0 kg/m^2^). Having formal education, being from a wealthier household and urban residence were associated with higher BMI and higher prevalence of overweight among women (Table 4). While the strength of these relationships varied by country, the directionality remained consistent. However, in the Maldives none of the three key exposure variables remained significantly associated with mean BMI; and education and wealth were not significantly associated with prevalence of overweight. In Pakistan, education was not significantly associated with mean BMI.

**Figure 2:**
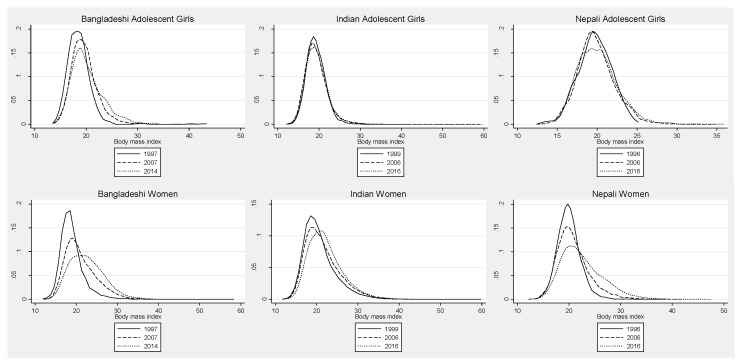
Distribution of body mass index (BMI) of adolescent girls and adult women in Bangladesh, India and Nepal over time.

The prevalence of overweight among women rose from 3% to 26% in Bangladesh (1996–2014), from 12% to 24% in India (1999–2016) and from 2% to 27% in Nepal (1996–2016), with distinct shifts in the distribution of BMI among women widening to the right over time in Bangladesh, India, and Nepal, corresponding to the higher mean BMI (Figure 2). While overweight and obesity increased in all three countries over time, we observed a slower rate of increase in overweight in Bangladesh and Nepal between the latter two surveys compared with earlier rates of change (Table S3). With regards to the prevalence of obesity, the rate of change increased in Bangladesh and India over the three survey periods and decreased in Nepal.

**Figure 3:**
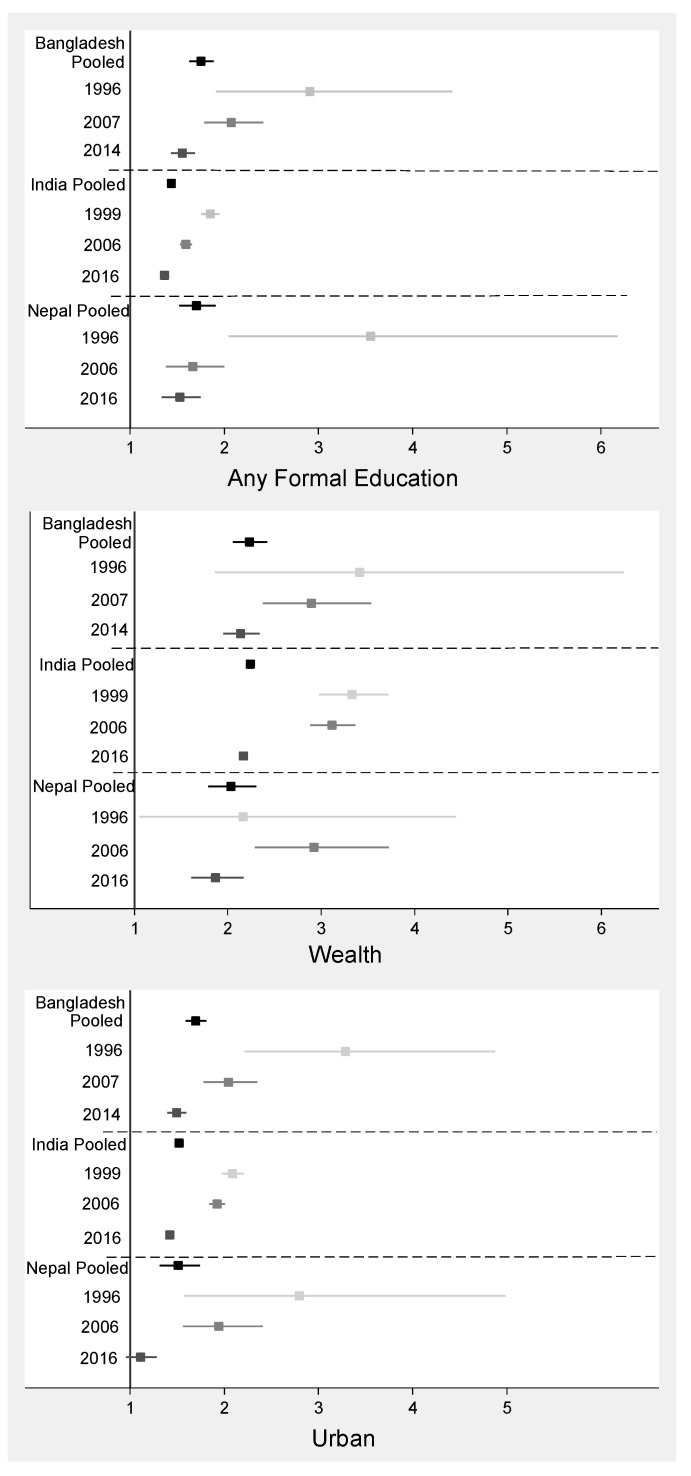
Adjusted Prevalence Ratios (95% CI) ^2^ for overweight ^1^ among women and three key factors in Bangladesh, India and Nepal, presented pooled (black squares) and stratified by survey year (light grey, 1990s time point; medium grey, early 2000s time point; dark grey, most recent time point in 2010s). ^1^ Overweight is defined as a body mass index ≥25.0 kg/m^2^. ^2^ Each adjusted prevalence ratio represents a separate modified Poisson model controlling for the other factors listed in the table, woman’s age, country-specific sub-region and cluster.

Survey year was a significant effect modifier of the relationship between each key exposure variable (education, wealth, and urban residence) and the prevalence of overweight among women in Bangladesh and India and modified the relationships between education and overweight and urban residence and overweight in Nepal (Table S4). These relationships tested between key factors (education, wealth, and urban residence) and overweight remained significant after the models were stratified by survey year (Figure 3). The prevalence of overweight rose over time, though the disparities by factors decreased over time point in each country, such that all adjusted prevalence ratios trended towards 1 over time.

## 4. Discussion

In our analytic sample comprising the most recent year of surveys across South Asia, approximately 2.6% of children aged 0–59 months in the sample were overweight, ranging from 1.3% in Nepal to 5.8% in the Maldives. While this is slightly lower than the estimated overall South Asia average for 2016 of 4.3% (2.4% to 7.5%), the rate of growth is certainly worrisome. The prevalence of overweight among children has increased considerably in South Asia since 1990, and it is likely to continue. In highlighting the two- to five-fold rise in overweight and obesity in Afghanistan, Bangladesh and India since the early 2000s, Aguayo and Paintal (2017) pointed out that the diet of adolescent girls and younger children remains poor, and that nutrition programmes do not focus on managing the seriousness of the double burden of malnutrition across South Asia [39].

### 4.1. Pre-School Aged Children

The literature presents some inconsistencies regarding the relationship between family history of obesity and child obesity in South Asia. For example, two independent studies among school-aged children in urban Bangladesh and affluent India each concluded that a family history of obesity increased children’s likelihood of being obese [40,41]. Yet, another study among affluent school-aged children in India found no significant association between child and parental obesity [42]. The current analysis provides clear and consistent results across countries of a significant association between maternal overweight and child WHZ as well as child overweight in South Asia. Furthermore, in a similar pooled analysis examining child wasting, maternal underweight was associated with child wasting, further supporting this relationship [27]. Prior research supports that both environmental and genetic factors may in part explain the association between maternal overweight and child WHZ and overweight. Maternal overweight and obesity puts women at a greater risk of pre-term delivery (<32 weeks gestation) [43,44,45]. Premature birth in turn increases children’s risk of morbidity, mortality, and cognitive deficits [12,46,47,48]. Furthermore, the metabolic consequences that result from premature birth can be exacerbated in growing obesogenic environments, for instance regions undergoing rapid urbanization and nutrition transition [12,49]. Thus, the mechanisms through which maternal overweight and obesity impact child WHZ and overweight, including epigenetics and fetal programming, need to be further explored.

MDD and MAD, two indicators of appropriate young child feeding practices, were positively associated with increased mean WHZ in children and with a higher prevalence of overweight; at the same time, MMF was only associated with increased mean WHZ and not with overweight. In a region where the mean WHZ in under-fives is significantly below the mean WHZ of a well-nourished reference population, the significant association of complementary feeding indicators with increased mean WHZ is encouraging. However, the association of MDD and MAD with overweight needs further exploration; it suggests that while achieving diet diversity as a policy goal typically aimed at tackling undernutrition, how it is achieved may matter in the context of risk factors for overweight and obesity. As the prevalence of childhood overweight and obesity rise, monitoring infant and young child feeding practices among different populations in relation to these outcomes will be important. For instance, breastfeeding is known to have protective effects against overweight and obesity and positive survival, growth and development outcomes universally [50,51]. In contrast, some parental feeding styles (i.e., indulgent or restrictive) are associated with child weight, although the specific parental feeding styles that are associated with child weight vary across contexts [52,53,54].

### 4.2. Adolescent Girls

Mean BMI among adolescent girls from the most recent year of surveys in this study ranged from 19.4 (±3.0) kg/m^2^ in India (2006) to 21.9 (±4.6) kg/m^2^ in the Maldives, and the prevalence of overweight ranged between 4.4% in Nepal and 24.5% in the Maldives. In our sample, the prevalence of overweight in adolescent girls was 4.6% using BMI ≥25.0 kg/m^2^ and 11.6% using BMI ≥23.0 kg/m^2^.

Overall, adolescent girls were more likely to be overweight if living in urban households, even controlling for wealth. This finding is consistent with the work of Jaaks et al. (2015) which considered the double burden through 53 Demographic and Health surveys globally; they found that 38% of urban areas had both an underweight and overweight prevalence exceeding 10% [55]. That said, there is ample evidence that overweight and obesity are increasing in rural as well as poorer households across Asia [1,39]. The rising prevalence of overweight in our Bangladesh sample is of particular concern since the prevalence among adolescents increased from <1% in 1996 to >8% in 2014; that is a rate of change of 0.16 percentage points per year in the first 10 years, followed by 0.14 percentage points per year in the last 7 years (2007–2014). The rise in obesity was steeper still, at 0.44 percentage points in the years up to 2014.

Our analysis, agreeing with that of Leroy et al. (2018) on Bangladesh, suggests that wealth and education are not consistently significant moderators of the rising burden of overweight among girls or adolescents [56]. In other words, while rapid poverty reduction across Bangladesh has driven a successful lowering of child stunting in recent decades, rising incomes and access to education have not prevented the concomitant acceleration in obesity outcomes.

That said, across our sample as a whole we found that formal education was not significantly associated with (i.e., not promoting) overweight among adolescent girls once stratified by country, despite the fact that higher education tends to be associated with more overweight and obesity elsewhere [14]. Ideally, formal education systems should be negatively correlated with both forms of malnutrition, but directly via improved knowledge of optimal nutrition and health practices, and indirectly via income-earning potential. South Asia’s education systems are in effect passive bystanders to the unfolding of a hugely significant nutrition and health crisis; they must do more to inform, educate and promote improved dietary choices, physical activity and healthy behaviours [39].

Finally, it has been highlighted that overweight and obesity among adolescent girls is inconsistently measured or reported, and that when reported reports often include different age groups and definitions for overweight and obesity [57]. This was evident across the surveys included in our sample, with some surveys reporting on all adolescent girls and, in most, the category of adolescent girls was extrapolated from the sample of women of reproductive age (15- to 49-year-olds), generating an important data gap among younger adolescent girls. Thus, South Asian countries need to collect nationally representative data on the nutrition of adolescent girls and boys aged 10–19 disaggregated by sex, age group, geographic region, and socioeconomic status. Data need to show the extent and severity of the triple burden of poor nutrition in adolescents: growth failure (stunting and wasting), micronutrient deficiencies and anaemia, and overweight and obesity. There must also be measures of the adequacy of adolescents’ diets, and the socioeconomic determinants affecting this. The effect of national policies and programmes for adolescent nutrition must be measured, including their coverage, effectiveness, and equity. This evidence should be used to develop national policies and to scale up cost-effective programmes [39].

### 4.3. Adult Women

Adult women in this sample had a mean BMI of 22.3 (±4.3) kg/m^2^ but 22% of the women were overweight with a BMI ≥25.0 kg/m^2^. India had the lowest prevalence of overweight among women in the region (24%), while the Maldives (46%) and Pakistan (41%) had levels that are significantly above the global estimate of overweight among women (38%) [1]. Women with some formal education, from wealthier homes and living in urban areas were more likely to be overweight. Similar findings have been reported for Sri Lanka among women aged 35 to 64 years [9]. Furthermore, the overall association between wealth and overweight in low and middle-income countries has been reported in the literature [58,59].

In our analysis, women’s mean BMI increased with survey year in Bangladesh, India and Nepal at rates between <0.001 and 0.011 kg/m^2^ per year; this is lower than the recent global estimate presented by Popkin et al. of 0.4 to 0.5 kg/m^2^ per year increase over past 3 decades [12]. The rate of change in overweight and obesity among Bangladeshi and Nepalese women in this study between the mid−2000 s and the 2010 s was approximately 0.1 percentage point and 0.14–0.18 percentage point per year, respectively, suggesting that obesity may be increasing at a faster rate than overweight. The rate of change in mean BMI, overweight and obesity appears to be lower in India compared with Bangladesh and Nepal, yet the most recent prevalence of both overweight and obesity are similar across these three countries.

The relationship between the key exposure variables evaluated in this study (formal education, household wealth, and urban residence) and the prevalence of overweight among women attenuated over time. On a global level, the gap in the prevalence of overweight between urban and rural women is narrowing, with the prevalence increasing at a quicker rate in rural areas compared with urban settings [12]. In a longitudinal analysis of cross-sectional data from China, Jones-Smith et al. reported similar findings to those of the current study; over time, the prevalence of overweight increased at a greater rate among women with lower education than among women with higher education [14]. A study assessing trends across 39 countries found that high socioeconomic status was associated with greater overweight prevalence among women, but that higher gross national product (GNP) was associated with a relatively greater overweight prevalence among women of lower socioeconomic status [10].

It is possible that the trends observed in Bangladesh, India, and Nepal are also related to their status as economies emerging from a history of pervasive poverty and limited economic growth. While the prevalence of overweight and obesity remain higher among more educated, wealthier urban women in South Asia, these findings suggest that attention should also be paid to overweight and obesity among those with less education, from poorer households and in rural areas, as the prevalence of overweight seems to be increasing more rapidly in these subgroups that are also more vulnerable to stunting, wasting, micronutrient deficiencies, and diet-related non-communicable diseases (NCDs).

There are some important risk factors associated with overweight that could not be evaluated in this study. A balance of energy intake and expenditure translate into weight loss, maintenance or gain, and several individual studies have documented the impact of dietary choices and physical activity on overweight and obesity [7,60,61]. As populations transition from labour-intensive jobs and high carbohydrate diets to more sedentary jobs and diets high in edible oils, sweeteners and animal source foods, diet-related non-communicable diseases tend to increase [12]. On a global level, Green et al. found a significant association between energy available from meat, dairy products and vegetable oils with higher ischemic health disease [62]. To address the growing concerns of overweight and obesity in South Asia, a critical examination of the food environment, access to good quality diets, and the effectiveness of food-based programming in relation to all forms of malnutrition is urgently needed.

Overweight has substantial health and economic costs to individuals and countries. Maternal overweight is associated with poor health outcomes and huge medical costs associated with gestational diabetes and pre-eclampsia, preterm birth and maternal and infant mortality [63]. Economically, costs can be broken down to direct medical and non-medical costs and indirect costs of morbidity and mortality [64]. One systematic review of economic costs has estimated that obese individuals had 30% greater medical costs compared to individuals with normal weight [65].

South Asia has the largest number of undernourished children, anemic women and small for gestational age births globally [63], all of which continue to have high social and economic implications on the region. The added burden of overweight and the associated implications are on the rise and require rapid policy and programme attention.

### 4.4. Limitations and Strengths

This study has several limitations and strengths. The data were aggregated from cross-sectional surveys and the survey weights from each dataset were not used in this pooled analysis, and thus should be interpreted as such. While we attempted to access data from all South Asian countries, similar data were not accessible from Bhutan and Sri Lanka at the time of the analysis. Furthermore, data on adolescent girls are lacking throughout the region. Standard DHS modules do not include an evaluation of the nutritional status of adolescents specifically, thus our sample of adolescent girls was limited to the 15- to 19-year-old group, which are included in the DHS standard survey module for “women of reproductive age”. For consistency, we used this age range from the Afghanistan survey as well.

This study highlights the importance of overweight as a growing public health issue in South Asia among pre-school age children, adolescent girls, and adult women. By pooling compatible datasets from national surveys across South Asia over time, we were able to examine which factors are significantly associated with overweight using a large sample.

The World Health Assembly has adopted six global nutrition targets for the year 2025, including the goal of “no increase in childhood overweight” [66]. This target was established because of the serious health risks associated with child overweight and obesity, which in turn often leads to adult overweight and obesity. Most countries in South Asia are currently off-target. In addition, there is a voluntary global target in the WHO Global Monitoring Framework for Non-Communicable disease to halt the rise in obesity by 2025 and a call to monitor non-communicable disease [67]. Policymakers in South Asia will need to urgently prioritize investments to reverse recent trends in overweight in children, adolescents and women and address a situation that is becoming of critical public health concern.

## 5. Conclusions

The prevalence of overweight in pre-school age children, adolescent girls and adult women continues to rise across South Asia. This represents a serious public health nutrition and development challenge that requires immediate policy attention in tandem with prevailing agendas that seek to resolve all forms of undernutrition. Multi-duty policies and programmes are urgently needed to ensure that the many faces of malnutrition are addressed simultaneously rather than in silos. According to a recent study of global trends, “the number of children and adolescents aged 5–19 years in the world who are moderately or severely underweight remains larger than those who are obese, showing the continued need for policies that enhance food security in low-income countries and households, especially in South Asia” [68]. Globally, the rise in adolescent overweight and obesity is larger than the decline in underweight. It has been estimated that if post-2000 trends continue unabated, the prevalence of child and adolescent obesity will surpass the prevalence rate of underweight by the year 2022 [68]. As a result, the dual nutrition crisis posed by undernutrition and overweight/obesity continues to grow.

Overweight is no longer a high income, wealthy household problem. Increasingly, overweight is recorded among poorer, rural, and less educated individuals and households, alongside individuals from wealthy, urban, and highly educated households. Indeed, undernutrition and overweight increasingly co-exist in the same countries, communities and families. Maternal overweight has adverse effects on birth outcomes and, as shown in our analysis, is associated with overweight among pre-school aged children. Innovative dual-duty policies and programmes are needed to address challenges posed by co-existing nutrition burdens [69]. The rising social and economic burdens posed by nutrition-related poor health and development can only be addressed by resolving poverty and income disparities and ensuring healthy diets for all, facilitating appropriate levels of physical activity, and implementing effective multi-sectoral actions that address the direct, underlying and distal causes of malnutrition in all its forms.

## Figures and Tables

**Table 1:** Descriptive statistics of the study population ^1,2^.

	**Afghanistan**	**Bangladesh**			**India**			**Maldives**	**Nepal**			**Pakistan**	**Recent Pooled ^3^**
	**2013**	**1997**	**2007**	**2014**	**1999**	**2006**	**2016**	**2009**	**1996**	**2006**	**2016**	**2013**	
Pre-school aged children													
*n*	10,958	4394	5022	6632	22,609	37,881	206,463	2226	3321	4809	2218	2624	231,121
Age (months)													
<6 months	16.9	10.8	9.2	8.5	19.2	9.2	8.9	9.8	17.7	10.2	9.8	11.2	9.5
6–12 months	15.7	12.8	12.9	14.1	20.8	12.5	12.7	14.7	22.7	11.7	13	12.2	12.8
13–24 months	19.5	20.1	21.3	21.5	32.3	19.1	19.4	20.6	33.8	19.1	20.7	17.4	19.4
25–36 months	18.1	18.7	19.7	19.7	27.7	19.5	19.7	19.7	25.9	20.5	18.6	20	19.6
37–48 months	17.4	20	18.8	19.2	0	20.3	20.9	18.8	0	20.1	19.9	19.4	20.6
49–59 months	12.4	17.6	18.2	17	0	19.4	18.5	16.5	0	18.5	18	19.8	18.1
Female	48.1	50.3	50.2	47.7	47.8	47.1	47.4	49.9	48	48	47.3	48.8	47.7
Urban	23.3	9.5	21.6	25.7	23.8	25.1	28.4	31	5.9	12.4	53.3	31	24.3
Weight-for-height *z*-score [mean ± SD]	−0.27 ± 1.42	−0.99 ± 1.33	−1.04 ± 1.08	−0.88 ± 1.16	−0.88 ± 1.44	−1.01± 1.29	−1.02 ± 1.37	−0.44 ± 1.41	−0.80 ± 1.19	−0.84 ± 1.07	−0.65 ± 1.14	−0.52 ± 1.30	−0.91 ± 1.40
Height-for-age *z*-score [mean ± SD]	−1.50 ± 1.76	−2.31 ± 1.53	−1.73 ± 1.35	−1.52 ± 1.31	−1.94 ± 1.77	−1.82 ± 1.66	−1.45 ± 1.67	−0.89 ± 1.39	−2.15 ± 1.40	−1.89 ± 1.34	−1.51 ± 1.34	−1.75 ± 1.72	− 1.46 ± 1.68
Stunting (HAZ < −2)	38.4	59.6	42.3	35.3	49.7	46.9	37.6	17.9	55.8	48.1	35.4	44.5	37.6
Wasting (WHZ < −2)	9.5	20.7	17.4	14.3	19.8	19.8	21.1	10.6	15	12.7	10.2	10.9	19.6
Overweight (WHZ > 2)	5.3	1.9	1	1.5	2.9	1.6	2.1	5.8	1	0.6	1.3	3.3	2.6
	**Afghanistan**	**Bangladesh**			**India**			**Maldives**	**Nepal**			**Pakistan**	**Recent Pooled ^3^**
	**2013**	**1997**	**2007**	**2014**	**1999**	**2006**	**2016**	**2009**	**1996**	**2006**	**2016**	**2013**	
Adolescent girls (15–19 years old)													
*n*	2231	668	1096	1667	5119	21,818	118,602	75	336	2282	1251	164	123,990
Age (years) [mean ± SD]	16.8 ± 1.4	17.2 ± 1.3	17.5 ± 1.3	17.5 ± 1.3	17.6 ± 1.3	16.9 ± 1.4	16.9 ± 1.4	18.7 ± 0.5	18.1 ± 1.0	16.9 ± 1.4	16.9 ± 1.4	18.1 ± 0.8	16.9 ± 1.4
Urban	30.3	8.1	17.4	26.5	14.3	30.1	30.3	24.5	5.3	14.6	63.5	22.8	27
Married	20.7	100	100	100	100	23.6	12.6	100	100	27.8	23.4	100	12.8
Number of times given birth [mean ± SD]	1.5 ± 0.8	1.3 ± 0.5	0.8 ± 0.7	0.7 ± 0.6	0.7 ± 0.8	0.1 ± 0.4	0.1 ± 0.3	0.2 ± 0.4	1.2 ± 0.5	0.2 ± 0.4	0.2 ± 0.4	0.5 ± 0.6	0.1 ± 0.3
No education	45.4	44.6	9.6	4.9	53.1	20.1	6.5	0	66.5	19.8	5.8	49.7	7.5
Short stature (<145 cm)	9.6	18.1	16.6	13	14.9	11.7	12.7	5.5	13.9	14.41	10.3	8.8	12.6
Body mass index (BMI) (kg/m^2^) [mean ± SD]	21.0 ± 3.2	18.6 ± 2.4	19.6 ± 2.4	20.2 ± 3.2	19.1 ± 2.3	19.0 ± 2.5	19.4 ± 3.0	21.9 ± 4.6	19.6 ± 2.1	19.9 ± 2.3	20.0 ± 2.6	20.6 ± 2.8	19.5 ± 2.9
Underweight (<18.5 kg/m^2^)	21.6	50.3	34.4	30.9	42.2	46.6	41.8	23.4	29.9	26.2	30	22.5	38.9
Overweight based on cut-off ≥23.0 kg/m^2^	20.4	2.3	8.2	17	4.5	6.1	9.5	37.5	5.2	10.1	12.3	17	9.5
Overweight based on cut-off ≥25.0 kg/m^2^	9.5	0.7	3	7.3	1.4	2.4	4.25	24.5	0	2.1	3.3	7.1	3.9
Obese (≥30.0 kg/m^2^)	1.9	0.7	0.1	1.3	0.1	0.2	0.8	2.8	0	0.04	0.4	0.4	0.7
*Based on IOTF standards*													
Underweight (<18.5 kg/m^2^)	16.7	46.6	30.8	26.7	38.1	39.1	35.1	23.4	28.8	18.8	23.8	22	32.4
Overweight based on cut-off ≥23.0 kg/m^2^	25.3	2.5	9.3	19.2	5.5	7.5	11.2	37.5	6.1	12.7	14.7	18.2	11.6
Overweight based on cut-off ≥25.0 kg/m^2^	10.4	0.7	3.2	8.2	1.6	2.7	4.9	24.5	0	3	4.4	7.1	4.6
Obese (≥30.0 kg/m^2^)	2	0.7	0.1	1.3	0.1	0.3	0.9	2.8	0	0.1	0.4	0.4	0.7
	**Afghanistan**	**Bangladesh**			**India**			**Maldives**	**Nepal**			**Pakistan**	**Recent Pooled ^3^**
	**2013**	**1997**	**2007**	**2014**	**1999**	**2006**	**2016**	**2009**	**1996**	**2006**	**2016**	**2013**	
Adult women (20–49 years old)													
*n*	9520	3377	9031	14,957	72,494	91,257	536,554	5138	2972	7834	4914	3969	575,052
Age (year)	31.0 ± 7.6	27.5 ± 5.9	32.7 ± 8.4	32.9 ± 8.3	32.8 ± 8.1	32.5 ± 8.2	33.1 ± 8.5	33.4 ± 8.0	28.0 ± 6.1	32.6 ± 8.5	32.7 ± 8.3	33.9 ± 8.3	33.0 ± 8.4
Urban	27.23	10.35	23.51	28.63	27.65	32.97	35.22	32.14	6.1	16.08	63.02	34.01	30
Number of times given birth [mean ± SD]	4.7 ± 2.5	3.6 ± 2.2	3.2 ± 2.0	2.8 ± 1.7	3.3 ± 2.1	2.8 ± 2.1	2.3 ± 1.7	2.9 ± 2.3	3.7 ± 2.2	3.2 ± 2.2	2.5 ± 1.8	3.9 ± 2.7	2.4 ± 1.8
No education	80.4	56.4	38.1	28	52.5	45.3	32.22	26.2	81.1	62.6	40.82	57.4	33.96
Short stature (<145 cm)	3.7	17.1	15	12.7	13	11.4	10.81	12.6	15	14.1	10.92	5	10.21
Body mass index (kg/m^2^) [mean ± SD]	23.5 ± 4.5	18.9 ± 2.9	20.8 ± 3.6	22.5 ± 4.2	20.4 ± 3.8	20.8 ± 4.1	22.4 ± 4.5	24.9 ± 4.7	19.8 ± 2.2	20.8 ± 3.2	22.7 ± 4.2	24.4 ± 5.6	22.3 ± 4.3
Underweight (<18.5 kg/m^2^)	9.1	51.6	28.9	17.2	35.7	32.7	18.81	7.2	27.5	23.7	13.98	13.4	18.19
Overweight based on cut-off ≥23.0 kg/m^2^	46.1	7.2	23.6	41.6	19.8	24.9	38.34	63.8	7.5	20.6	40.99	55.8	36.64
Overweight based on cut-off ≥25.0 kg/m^2^	29.3	3.2	12.9	25.6	11.4	15.1	24.09	45.8	1.9	10.4	26.81	41.2	22.24
Obese (≥30.0 kg/m^2^)	8.7	0.6	1.9	4.7	2.4	3.5	6.04	13.2	0.1	1.2	6.26	15.4	5.25

^1^ Percent of the sample unless otherwise specified; ^2^ Values are based on sample weights from the survey for each column; ^3^ Values are not based on sample weights. WHZ, weight-for-height *z*-score.

**Table 2:** Child weight-for-height *z*-score (WHZ) and prevalence of overweight ^1^ among children with mothers who are overweight ^2^ or not.

	**Overweight Mothers**			**Not Overweight Mothers**				
	***n***	**Mean WHZ**	**SD**	***n***	**Mean WHZ**	**SD**	**Adj. β ^3^**	**SE**
Maternal overweight in relation to mean WHZ								
Pooled	34,193	−0.55	1.37	194,771	−0.98	1.40	0.29 **	0.01
Afghanistan	2161	−0.17	1.45	7546	−0.35	1.47	0.15 **	0.04
Bangladesh	1262	−0.57	1.17	5348	−0.96	1.13	0.28 **	0.04
India	28,715	−0.59	1.36	176,992	−1.02	1.39	0.28 **	0.01
Maldives	871	−0.42	1.43	1245	−0.57	1.38	0.16 *	0.06
Nepal	326	−0.23	1.12	1889	−0.71	1.11	0.38 **	0.08
Pakistan	858	−0.11	1.45	1751	−0.44	1.49	0.34 **	0.06
	**Overweight Mothers**			**Not Overweight Mothers**				
	***n***	**Overweight (%)**		***n***	**Overweight (%)**		**AOR ^4^**	**SE**
Maternal overweight in relation to overweight among children								
Pooled	34,193	3.57		194,771	2.35		1.34 **	0.05
Afghanistan	2161	6.57		7546	5.13		1.27	0.19
Bangladesh	1262	2.38		5348	1.07		1.51	0.39
India	28,715	3.17		176,992	2.24		1.22 **	0.05
Maldives	871	6.2		1245	4.34		1.56 *	0.32
Nepal	326	3.99		1889	0.95		5.19 **	2.49
Pakistan	858	8.16		1751	5.6		1.87 **	0.4

* *p* < 0.05; ** *p* < 0.01; SD, standard deviation; SE, standard Error; AOR, adjusted odds ratio; ^1^ Child overweight is defined as WHZ >2; ^2^ Mother’s overweight is defined as BMI ≥25 kg/m^2^; ^3^ Each adjusted beta coefficient represents a separate multivariate mixed linear regression model controlling for child’s age and sex, mothers age and education, wealth index, urban and clusters within countries. Country-specific models additionally include a variable for the country-specific sub-regions; ^4^ Each adjusted odds ratio represents a separate multivariate mixed logistic regression model controlling for child’s age and sex, mother’s age and education, wealth index, urban and clusters within countries. Country-specific models additionally include a variable for the country-specific sub-regions.

**Table 3:** Child weight-for-height *z*-score (WHZ) and prevalence of overweight ^1^ among children 6 to 23 months of age who met specific infant and young child feeding recommendations and those who did not.

	**Yes**			**No**				
	***n***	**Mean WHZ**	**SD**	***n***	**Mean WHZ**	**SD**	**Adj. β ^2^**	**SE**
Feeding practices in relation to mean WHZ ^2^								
Minimum meal frequency	24,906	−0.84	1.45	40,826	−0.98	1.47	0.05 **	0.01
Minimum diet diversity	15,245	−0.74	1.46	72,916	−0.99	1.57	0.12 **	0.01
Minimum acceptable diet	6784	−0.72	1.45	58,167	−0.96	1.47	0.08 **	0.02
		**Yes**		**No**				
	***n***	**% Overweight**		***n***	**% Overweight**		**AOR ^3^**	**SE**
Feeding practices in relation to % overweight ^3^								
Minimum meal frequency ^1^	24,906	3		40,826	2.76		1.05	0.06
Minimum diet diversity	15,245	3.35		72,916	3.66		1.28 **	0.07
Minimum acceptable diet	6784	3.27		58,167	2.76		1.19 *	0.1

* *p* < 0.05; ** *p* < 0.01; SD, standard deviation; SE, standard Error; AOR, adjusted odds ratio; ^1^ Overweight is defined as WHZ >2; ^2^ Each adjusted beta coefficient represents a separate multivariate mixed linear regression model controlling for child’s age and sex, mothers age and education, wealth index, urban and clusters within countries. Because these relationships did not differ by country, the models were not stratified; ^3^ Each adjusted odds ratio represents a separate multivariate mixed logistic regression model controlling for child’s age and sex, mothers age and education, wealth index, urban and clusters within countries. Because these relationships did not differ by country, the models were not stratified.

**Table 4:** Relationship between girls’ and women’s body mass index (BMI) and prevalence of overweight ^1^ with three key factors, presented pooled and stratified by country.

**Adolescent Girls**																					
	**Pooled**					**Stratified by Country ^4^**															
						**AFG**		**BGD**			**IND**			**MDV**			**NPL**			**PAK**	
	***n***	**Mean BMI**	**SD**	**Adj. β ^2^**	**SE**	**Adj. β**	**S**	**Adj. β**	**SE**		**Adj. β**	**SE**		**Adj. β**	**SE**		**Adj. β**	**SE**		**Adj. β**	**SE**
Factors in relation to mean BMI																					
Any education	114,704	19.5	2.9	0.05	0.03	−0.15	0.14	0.42	0.35		0.01	0.03		--	--		0.24	0.36		−0.42	0.53
Wealthier	68,431	19.8	3.2	0.47 **	0.02	0.37 *	0.16	0.93 **	0.17		0.39 **	0.02		1.35	1.15		0.11	0.16		2.11 **	0.6
Urban	33,469	19.9	3.3	0.36 **	0.02																
	**Pooled**					**Stratified by Country ^4^**															
						**AFG**		**BGD**			**IND**			**MDV**			**NPL**			**PAK**	
	***n***	**% Overweight**		**AOR ^3^**	**SE**	**AOR**	**SE**	**AOR**	**SE**		**AOR**	**SE**		**AOR**	**SE**		**AOR**	**SE**		**AOR**	**SE**
Factors in relation to % overweight																					
Any education	114,704	4.73		1.22 **	0.08	1.01	0.17	1.81	1.16		1.18	0.09		--	--		1.62	1.79		1.28	0.94
Wealthier	68,431	6.54		2.46 **	0.09	1.42	0.3	2.73 **	0.71		2.27 **	0.09		3.08	2.38		2.07*	0.7		16.68 *	20.9
Urban	33,469	7.68		1.74 **	0.06	1.17	0.26	1.52	0.35		1.77 **	0.06		1.11	1.28		0.97	0.33		0.54	0.43
**Women**																					
	**Pooled**					**Stratified by Country ^4^**															
						**AFG**		**BGD**			**IND**			**MDV**			**NPL**			**PAK**	
	***n***	**Mean BMI**	**SD**	**Adj. β ^2^**	**SE**	**Adj. β**	**SE**	**Adj. β**	**SE**		**Adj. β**	**SE**		**Adj. β**	**SE**		**Adj. β**	**SE**		**Adj. β**	**SE**
Factors in relation to mean BMI																					
Any education	379,713	22.62	4.35	0.85 **	0.01	0.45 **	0.11	1.14 **	0.08		0.83 **	0.01		0.04	0.18		1.03 **	0.13		0.35	0.19
Wealthier	350,054	23.23	4.45	1.50 **	0.01	0.36 **	0.1	1.64 **	0.08		1.41 **	0.01		−0.01	0.15		1.27 **	0.13		1.32 **	0.22
Urban	172,480	23.63	4.66	1.20 **	0.02	1.53 **	0.15	1.34 **	0.1		1.16 **	0.02		0.62	0.32		0.41 **	0.16		1.73 **	0.23
	**Pooled**					**Stratified by Country ^4^**															
						**AFG**		**BGD**			**IND**			**MDV**			**NPL**			**PAK**	
	***n***	**% Overweight**		**APR ^3^**	**SE**	**APR**	**SE**	**APR**	**SE**		**APR**	**SE**		**APR**	**SE**		**APR**	**SE**		**APR**	**SE**
Factors in relation to % overweight																					
Any education	379,713	25.04		1.36 **	0.01	1.15 **	0.06	1.56 **	0.06		1.37 **	0.01		1.07	0.04		1.53 **	0.1		1.12 *	0.05
Wealthier	350,054	29.89		2.20 **	0.02	1.18 **	0.06	2.14 **	0.1		2.17 **	0.02		1	0.04		1.87 **	0.14		1.52 **	0.1
Urban	402,565	33.49		1.41 **	0.01	1.60 **	0.09	1.49 **	0.05		1.42 **	0.01		1.20 *	0.09		1.11 **	0.08		1.29 **	0.06

* *p* < 0.05; ** *p* < 0.01; AFG, Afghanistan; BGD, Bangladesh; IND, India; MDV, Maldives; NPL, Nepal; PAK, Pakistan, SD, standard deviation; SE, standard Error; AOR, adjusted odds ratio; APR, Adjusted Prevalence Ratio; ^1^ Overweight is defined as a body mass index ≥25.0 kg/m^2^ based on the International Obesity Task Force (IOTF) sex and age-adjusted values for adolescents and as a body mass index ≥25.0 kg/m^2^ for women; ^2^ Each adjusted beta coefficient represents a separate multivariate mixed linear regression model controlling for the other factors listed, woman’s age and clusters within countries. Country-specific models additionally include a variable for the country-specific sub-regions. Corresponding adjusted beta coefficients and standard errors are reported; ^3^ Each adjusted odds ratio represents a separate modified Poisson model controlling for the other factors listed, woman’s age and clusters within countries. Country-specific models additionally include a variable for the country-specific sub-regions. Corresponding adjusted prevalence ratios and standard errors are reported; ^4^ Model was stratified by country when country was a significant (*p* < 0.1) effect modifier of the relationship; ^5^ There were no adolescent girls from Maldives with no education.

## Supplementary Materials

The following are available online at https://www.mdpi.com/2072-6643/11/8/1899/s1, Table S1: Child weight-for-height *z*-score (WHZ) of children with mothers who are overweight versus those who are not; multiple years of surveys from Bangladesh, India and Nepal; Table S2: Relationship between prevalence of overweight ^1^ among adolescent girls and three key factors in Bangladesh, India and Nepal, presented pooled and stratified by survey year; Table S3: Rate of change ^1^ in child weight-for-height *z*-score (WHZ) and prevalence of overweight, and adolescent and women body mass index (BMI), prevalence of overweight and prevalence of obese in Bangladesh, India and Nepal across survey years.
