# Supplementary material for: Trends and Correlates of Overweight among Pre-School Age Children, Adolescent Girls, and Adult Women in South Asia: An Analysis of Data from Twelve National Surveys in Six Countries over Twenty Years
Source: Nutrients. 2019 Aug 14;11(8):1899. doi: 10.3390/nu11081899 (PMC6722576; doi:10.3390/nu11081899)
Supplement: Supplementary file 1 [file nutrients-11-01899-s001.pdf]

SUPPLEMENTAL MATERIAL

Supplemental Table 1. Child weight-for-height z-score (WHZ) of children with mothers who are overweight versus those who are not; multiple years of surveys from Bangladesh, India and Nepal

	Overweight mothers			Not overweight mothers				
	n	mean WHZ	SD	n	mean WHZ	SD	Adj. β[2]	SE
Bangladesh								
Pooled[1]	1,832	-0.56	1.2	14,109	-1	1.18	0.31**	0.03
1997	115	-0.62	1.53	4,210	-0.98	1.33	0.15	0.13
2007	455	-0.5	1.19	4,551	-1.08	1.07	0.42**	0.06
2014	1,262	-0.57	1.17	5,348	-0.96	1.13	0.28**	0.04
India								
Pooled	33,700	-0.57	1.36	232,197	-0.99	1.39	0.39**	0.04
1999	1,264	-0.25	1.38	21,274	-0.81	1.47	0.34**	0.04
2006	3,721	-0.46	1.3	33,931	-0.96	1.33	0.30**	0.02
2016	28,715	-0.59	1.36	176,992	-1.02	1.39	0.29**	0.01
Nepal								
Pooled	627	-0.2	1.11	9,701	-0.82	1.11	0.44**	0.05

*p<0.05; **p<0.01

[1]Each row represents a separate multivariate mixed linear regression model controlling for child’s age and sex, mothers age and education, wealth index, urban, country-specific sub-regions and clusters

[2]Adjusted beta coefficient for maternal overweight

[3]Models were stratified by survey year when year was a significant effect modifier in the relationship

Supplemental Table 2. Relationship between prevalence of overweight[1] among adolescent girls and three key factors in Bangladesh, India and Nepal, presented pooled and stratified by survey year

	N	% overweight	APR[2]	SE
Bangladesh				
No education	487	1.85	Ref.	
Some education	2944	5.81	0.99	0.37
Poor	1346	2.23	Ref.	
Not poor	2085	7.19	3.04**	0.7
Rural	2506	3.95	Ref.	
Urban	925	8.76	1.57*	0.3
1997	668	0.75	Ref.	
2007	1096	3.28	4.02**	1.92
2014	1667	8.34	12.52**	5.82
	N	% overweight	APR[2]	SE
India				
No education	13,766	1.93	Ref.	
Some education	131,772	4.51	1.31**	0.09
Poor	61,717	2.02	Ref.	
Not poor	83,822	5.93	2.29**	0.09
Rural	103,672	3.1	Ref.	
Urban	41,867	7.17	1.89**	0.06
1999	5119	1.8	Ref.	
2006	21818	3.78	1.37	0.16
2016	118602	4.46	2.26	0.26
	N	% overweight	APR[2]	SE
Nepal				
No education	493	1.62	Ref.	
Some education	3,040	3.88	1.67	0.76
Poor	1,437	2.3	Ref.	
Not poor	2,096	4.44	1.76*	0.42
Rural	2,119	2.74	Ref.	
Urban	1,414	4.81	1.4	0.3

1996			4	--
2006	2282	3.11	Ref.	
2016	1,251	4.4	1.51	0.32

*p<0.05; **p<0.01

[1]Overweight is defined as a body mass index ≥ 25.0 kg/m^2 based on the IOTF sex and age-adjusted values

[2]Each adjusted prevalence ratio represents a separate modified Poisson model controlling for the over factors listed in the table, woman's age, country-specific sub-region and cluster

[3]Model was stratified by survey year when survey year was a significant (p<0.1) effect modifier of the relationship

[4]No adolescent girls were overweight in this survey

Supplemental table 3. Rate of change[1] in child weight-for-height z-score (WHZ) and prevalence of overweight, and adolescent and women body mass index (BMI), prevalence of overweight and prevalence of obese in Bangladesh, India and Nepal across survey years

	Bangladesh			India			Nepal		
	1997	2007	2014	1999	2006	2016	1996	2006	2016
Children									
Mean WHZ	-0.99	-1.04	-0.88	-0.88	-1.01	-1.02	-0.8	-0.84	-0.65
Rate of change		0	-0.02		0.02	0.001		0	-0.025
Prevalence overweight (%)	1.9	1	1.5	2.9	1.6	2.1	1	0.6	1.3
Rate of change		-0.06	0.06		-0.07	0.03		-0.05	0.08
Adolescent girls									
Mean BMI (kg/m2)	18.6	19.6	20.2	19.1	19	19.4	19.6	19.9	20
Rate of change		0.01	0		0	0.002		0	0.001
Prevalence overweight[2] (%)	0.7	3.2	8.2	1.6	2.7	4.9	0	3	4.4
Rate of change		0.16	0.14		0.07	0.06		3	0.04
Prevalence obese[4] (%)	0.7	0.1	1.3	0.1	0.3	0.9	0	0.1	0.4
Rate of change		-0.18	0.44		0.15	0.12		3	0.15
Women									
Mean BMI (kg/m2)	18.9	20.8	22.5	20.4	20.8	22.4	19.8	20.8	22.7
Rate of change		0.01	0.01		0	0.007		0	0.009
Prevalence overweight[5] (%)	3.2	12.9	25.6	11.4	15.1	24.1	1.9	10.4	26.8
Rate of change		0.15	0.1		0.04	0.05		0.19	0.1
Prevalence obese[6] (%)	0.6	1.9	4.7	2.4	3.5	6.04	0.1	1.2	6.26

Rate of change		0.12	0.14		0.05	0.06		0.28	0.18

[1]Rate of change since previous time point

[2]Overweight among adolescents is defined as a body mass index ≥ 25.0 kg/m^2 based on the IOTF sex and age-adjusted values

[3]Could not be calculated as the prevalence at the previous time point was 0

[4]Obese among adolescents is defined as a body mass index ≥ 30.0 kg/m^2 based on the IOTF sex and age-adjusted values

[5]Overweight among women is defined as a body mass index ≥ 25.0 kg/m^2

[6]Obese among women is defined as a body mass index ≥ 30.0 kg/m^2

Supplemental Table 4. Relationship between prevalence of overweight[1] among adult women and three key factors in Bangladesh, India and Nepal, presented pooled and stratified by survey year

	Pooled				Stratified by survey year[3]											
					1996				2007				2014			
	N	% overweight	APR[2]	SE	N	% overweight	APR[2]	SE	N	% overweight	APR[2]	SE	N	% overweight	APR[2]	SE
Bangladesh																
No education	9,029	10.31	Ref.		1,859	1.29	Ref.		3,214	7.28	Ref.		3,956	17.01	Ref.	
Some education	18,336	24.45	1.74**	0.06	1,518	5.8	2.91**	0.62	5,817	19.29	2.08**	0.16	11,001	29.76	1.56**	0.06
Poor	9,911	8	Ref.		1,405	0.71	Ref.		3,056	4.02	Ref.		5,450	12.11	Ref.	
Not poor	17,454	26.48	2.24**	0.09	1,972	5.17	3.41**	1.05	5,975	20.64	2.90**	0.29	9,507	34.57	1.49**	0.05
Rural	18,098	13.64	ref.		2,881	2.05	ref.		5,503	8.54	ref.		9,714	19.96	ref.	
Urban	9,267	31.8	1.67**	0.05	496	10.69	3.29**	0.66	3,528	25.11	2.04**	0.14	5,243	38.3	1.49**	0.05
1997	3,377	3.32	ref.													
2007	9,031	15.01	2.91**	0.32												
2014	14,957	26.39	5.35**	0.58												

	Pooled				Stratified by survey year[3]											
					1999				2006				2016			
	N	% overwei	APR[2]	SE	N	% overw	APR[2]	SE	N	% over	APR[2]	SE	N	% over	APR[2]	SE

		ght				eight				weight				weight		
India																
No education	245,858	13.75	Ref.		35,563	6.2	Ref.		32,764	10.34	Ref.		177,531	15.9	Ref.	
Some education	454,428	23.92	1.44**	0.01	36,912	19.57	1.85**	0.05	58,493	22.58	1.59**	0.03	359,023	24.6	1.37**	0.01
Poor	255,124	8.45	Ref.		22,180	2.08	Ref.		22,847	3.6	Ref.		210,097	9.65	Ref.	
Not poor	445,181	27.17	2.24**	0.02	50,314	17.82	3.33**	0.19	68,410	23.06	3.12**	0.12	326,457	29.48	2.17**	0.02
Rural	475,163	15.18	Ref.		48,829	7.03	Ref.		49,620	10.3	Ref.		376,714	16.88	Ref.	
Urban	225,142	31.26	1.53**	0.01	23,665	25.34	2.09**	0.06	41,637	27.58	1.92**	0.04	159,840	33.09	1.42**	0.01
1999	72,494	13.01	Ref.													
2006	91,257	18.19	1.19**	0.02												
2016	536,554	21.71	1.72**	0.03												
	Pooled				Stratified by survey year[3]											
					1996				2006				2011			
	N	% overweight	APR[2]	SE	N	% overweight	APR[2]	SE	N	% overweight	APR[2]	SE	N	% overweight	APR[2]	SE
Nepal																
No education	9,350	7.91	Ref.		2,410	1.29	Ref.		4,870	6.88	Ref.		2,070	18.07	Ref.	
Some education	6,370	19.54	1.70**	0.1	562	5.52	3.56**	1	2,964	13.93	1.66**	0.16	2,844	28.16	1.53**	0.1
Poor	6,421	5.92	Ref.													

Not poor	9,299	17.26	2.03**	0.13												
Rural	10,113	7.09	ref.		2,718	1.62	ref.		5,648	6.41	ref.		1,747	17.8	ref.	
Urban	5,607	22.61	1.51**	0.11	254	7.09	2.80**	0.82	2,186	17.66	1.94**	0.21	3,167	27.28	1.11	0.08
1996	2,972	2.09	ref.													
2006	7,834	9.55	2.36**	0.35												
2016	4,914	23.91	6.05**	0.95												

*p<0.05; **p<0.01

[1]Overweight is defined as a body mass index ≥ 25.0 kg/m^2

[2]Each adjusted prevalence ratio (APR) represents a separate modified Poisson model controlling for the over factors listed in the table, woman's age, country-specific sub-region and cluster

[3]Model was stratified by survey year when survey year was a significant (p<0.1) effect modifier of the relationship
